# Combined effects of obesity and type 2 diabetes contribute to increased breast cancer risk in premenopausal women

**DOI:** 10.1186/1475-2840-8-33

**Published:** 2009-06-23

**Authors:** Majed S Alokail, Nasser M Al-Daghri, Omar S Al-Attas, Tajamul Hussain

**Affiliations:** 1 Department of Biochemistry, College of Science, King Saud University PO Box 2455, Riyadh 11451, Kingdom of Saudi Arabia

## Abstract

**Background:**

Both obesity and type 2 diabetes are among the risk factors for breast cancer development. Combined effect of these metabolic abnormalities on breast cancer risk however, has not been examined in premenopausal women. We tested this association in type 2 diabetic women, categorized as obese, overweight and normal body weight groups based on BMI.

**Design and methods:**

A total of 101 subjects were included in this study. Serum levels of IL-6, TNF-α, C reactive protein, leptin, TGF-α, adiponectin and insulin were measured by ELISA. Data were logarithmically transformed for variables not normally distributed. Analysis of variance with post-hoc Bonferroni was applied to compare the data between the groups. Simple and partial correlation coefficients between the variables were determined and a stepwise multiple linear regression analysis was performed to determine the relationships between the variables of interest.

**Results:**

Significantly increased levels of IL-6, C reactive protein, leptin and significantly decreased levels of adiponectin were found in obese group, while the levels of TNF-α and TGF-α were unaltered. A positive correlation between waist circumference and IL-6 was found in obese group. Similarly, C reactive protein, waist and hip circumferences were linearly correlated with BMI in obese group. Stepwise multiple linear regression analysis revealed several significant predictors for breast cancer risk.

**Conclusion:**

Obesity and type 2 diabetes, owing to their effects on adipocytokines and inflammatory mediators, contribute to increased breast cancer risk in premenopausal women. This study emphasizes healthy life style and better management of these metabolic disorders to avoid the pathogenesis of breast cancer and of other chronic diseases.

## Background

Obesity and type 2 diabetes mellitus (T2DM) remained major public health issues in the developed nations and their incidence is on the rise in the developing countries, attributed mainly to a considerable shift in the dietary practices [1,2]. On the other hand, breast cancer is the leading malignancy of women in industrialized nations and its incidence as well is increasing in developing countries [3]. Genetic predisposition and environmental factors such as high fat diet and alcohol consumption accompanied with sedentary life style constitute increased breast cancer risk. Thus, metabolic abnormalities including obesity and T2DM are positively associated with the breast cancer risk [4,5]. These conditions induce changes in several hormonal systems, including insulin, insulin like growth factor-1 (IGFl), estrogen, cytokines and growth hormones. A strong association was found between increased insulin resistance and postmenopausal as well as premenopausal breast cancer [6,7], while the increased estrogen levels are positively correlated with the postmenopausal breast cancer [8].

Obesity and T2DM also have in common an altered production of adipocytokines including leptin and adiponectin [9,10]. It has been shown that increased leptin levels and decreased adiponectin levels promote the carcinogenesis of the breast [11,12]. In addition, obesity is being increasingly recognized as sub clinical inflammation and accordingly there is an increased adipose tissue infiltration of inflammatory components including interleukin-6 (IL-6), C-reactive protein (CRP) and tumor necrosis factor-alpha (TNF-α), which have all shown to positively impact the breast cancer etiology [13-15]. Increased IL-6 levels in obesity can directly interfere with the insulin signaling and contribute to insulin resistance [16]. IL-6 controls the hepatic production of CRP, which is a risk factor for insulin resistance, breast cancer and cardiovascular diseases. Moreover, there is a positive correlation between IL-6 levels and circulating CRP [17], thus increased levels of IL-6 in obesity can directly or indirectly contribute to the risk to develop multiple chronic diseases. CRP is positively and negatively correlated with leptin and adiponectin levels respectively [18,19]. This is of special interest because altered levels of leptin and adiponectin are consistent with the elevated breast cancer risk in addition to the independent effect of CRP in enhancing the breast cancer risk [20]. Deletion of TNF-α or its receptor resulted in significantly improved insulin sensitivity in both obese mice and leptin deficient ob/ob mice [21]. In humans, adipose tissue TNF-α expression correlated with BMI, percentage of body fat and hyperinsulinemia, whereas weight loss decreased TNF-α, underlining the role of this cytokine in obesity induced insulin resistance [22]. TNF-α and IL-6 are also shown to stimulate the breast tumor growth in vivo [23]. Serum levels of C-peptide, a subunit of insulin that provides a clinically useful indication of insulin production and the degree of insulin resistance, have been associated with an increased breast cancer risk [24]. Additionally, HDL-cholesterol, hypertension, IGF, and hyperglycemia have all been found to be associated with breast cancer risk [7,25-28]. Transforming growth factor-alpha (TGF-α) is a member of the epidermal growth factor (EGF) family and it induces a mitogenic response by activating the EGF receptor tyrosine kinase [29]. Overexpression of TGF-α and its cognate receptor EGF-R have been implicated in the pathogenesis of breast cancer [30].

While majority of studies have indicated an inverse or absence of association of T2DM or obesity with premenopausal breast cancer risk, to our knowledge, none of the studies has examined the combined effects of these metabolic abnormalities in premenopausal women clinically asymptomatic for breast cancer. We tested this association in type 2 diabetic premenopausal women after categorizing them as normal body weight, overweight and obese groups based on BMIs.

## Methods

The subjects with T2DM were recruited from the roster of diabetic patients attending the outpatient unit of the Diabetes Center, King Abdulaziz University Hospital, Riyadh, Saudi Arabia. A total of 235 subjects were contacted for their willingness to participate in the study. Of these, 108 subjects provided their consents. From this cohort, 7 subjects were excluded from the study for the existence of clinical complications other than T2DM to avoid the misinterpretation of the data. Thus, this cross sectional study included 101 Saudi women clinically diagnosed with T2DM. Subjects were categorized based on BMI as normal weight, overweight and obese groups. Written and informed consents as well as completed generalized questionnaire were obtained from all the participants prior to their inclusion in the study. This study was conducted in accordance with the guidelines set by the ethics committee of the King Saud University, Riyadh, Saudi Arabia.

### Clinical measurements

Anthropometric measurements with emphasis to clinical markers of adiposity were obtained. BMI was calculated by dividing the weight (kg) with height (m^2^). Waist (cm) and hip (cm) circumferences were measured and waist-to-hip ratio was determined. Blood pressure (mm. Hg) was measured twice after 30 minutes of rest using the conventional mercurial sphygmomanometer and the values of the readings were averaged.

### Biochemical Parameters

Fasting blood glucose, and complete lipid profile (triglycerides, total cholesterol, LDL- and HDL-cholesterol) were determined using routine laboratory methods. Insulin was analyzed by a solid phase enzyme amplified sensitivity immunoassay (Medgenix INS-ELISA, Biosource, Belgium). Serum levels of leptin, TNF-α, CRP, IL-6 and adiponectin were measured by ELISA (R&D systems, MN. U.S.A. and Linco Research Inc, St.Charles, MO). TGF-α was measured using immunological techniques (Linco Research Inc, St. Charles, MO). Homeostasis model assessment (HOMA) was applied to evaluate fasting insulin resistance using the formula; glucose (mmol/L) × insulin (u μl/mL)/22.5.

### Statistics

SPSS version 11.5 for windows (Chicago, Illinois) was employed for statistical analysis of the data. Frequencies were presented in percent (%) of cases. Data was logarithmically transformed for variables, including insulin, TGF-α, triglycerides, leptin, CRP, IL-6 and HOMA-IR, for not being normally distributed. Analysis of variance with post-hoc Bonferroni was applied to compare the data between the groups. Simple and partial correlation coefficients between the variables were determined and a stepwise multiple linear regression analysis was performed to determine relationships between the variables of interest using IL-6 as the dependent variable and all other parameters as independent variables. Significance was set at P-value < 0.05.

## Results and discussion

**Figure 1:**
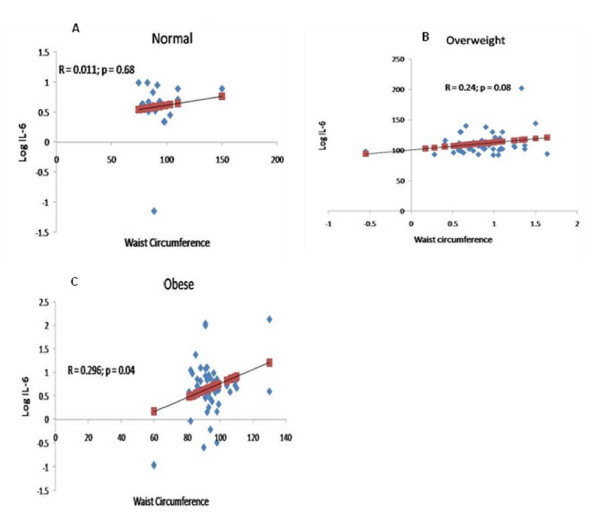
**Linear regression analyses between waist circumference and IL-6**. Linear regression analyses of IL-6 and waist circumference in normal body weight, overweight and obese groups revealed a significant positive correlation (P < 0.04; R = 0.296) in obese group.

Women who were obese were significantly older and had elevated systolic blood pressure, waist and hip circumferences (p < 0.05 respectively) as compared to women with normal BMI (Table 1). Among the metabolic parameters, obese women had significantly higher levels of serum leptin, CRP, IL-6 (p < 0.05 respectively) and significantly lower levels of adiponectin (p < 0.05) as compared to normal BMI group (Table 1). We found a significant positive correlation (R = 0.296; *P*< 0.04) between IL-6 and waist circumference in obese group (Figure 1C), while the correlation was insignificant in normal body weight (R = 0.011; *P*< 0.68) group as well as in overweight group (R = 0.24; *P*< 0.08) (Figure 1A and 1B). BMI was significantly correlated to waist and hip circumference as well as CRP levels in obese group and was insignificant in normal weight as well as overweight groups (Table 2). The rest of the associations were non-contributory.

**Table 1:** Clinical and metabolic parameters of the subjects

Parameters	Normal weight (N = 22)	Overweight (N = 34)	Obese (N = 34)
Age (years)	38.38 ± 11.54	44.71 ± 6.15	44.13 ± 8.42*

BMI (kg/m^2^)	22.54 ± 2.34	27.73 ± 2.21*	35.76 ± 3.48

Systolic BP (mmHg)	119.86 ± 14.26	127.53 ± 29.81	130.63 ± 15.78*

Diastolic BP (mmHg)	78.76 ± 9.15	76.82 ± 10.58	82.67 ± 12.77

Waist (cm)	90.0 ± 8.98	91.45 ± 8.52	107.57 ± 10.21*

Hips (cm)	92.86 ± 7.31	97.48 ± 8.23	107.0 ± 14.24*

Glucose (mmol/L)	7.28 ± 3.4	8.49 ± 3.86	8.13 ± 4.1

Insulin (μIU/ml)#	15.19 (1.83–42.75)	16.2 (0.51–42.25)	18.82 (2.45–55.82)

HOMA-IR #	5.46 (0.33–22.23)	5.91 (0.17–14.68)	7.0 (0.19–43.66)

Total cholesterol (mmol/L)	4.93 ± 0.92	4.84 ± 1.07	4.7 ± 1.3

LDL-cholesterol (mmol/L)	2.96 ± 0.75	2.77 ± 0.71	2.81 ± 1.01

HDL-cholesterol (mmol/L)	1.26 ± 0.34	1.22 ± 0.37	1.22 ± 0.32

Triglycerides (mmol/L)#	1.33 (0.5–3.04)	1.79 (0.43–7.41)	2.01 (0.43–20.49)

Leptin (ng/ml)#	16.4 (3.2–45.90)	24.86 (1.27–77.60)	35.28 (4.04–120)*

Adiponectin (μg/ml)#	7.6 (2.39–16.90)	4.6 (1.72–9.39)*	4.7 (1.9–7.95) *

CRP (μg/ml)#	2.3 (0–13.30)	5.2 (0–205.0)	6.9 (0.07–34.20)*

TNF-α (pg/ml)	4.8 ± 1.88	4.7 ± 2.0	4.5 ± 1.6

IL-6 (pg/ml)#	4.6 (0.07–10.34)	4.9 (0.11–24.1)	8.3 (0.28–45.30)*

TGF-α (pg/ml)#	13.8 (7.9–42.34)	11.6 (7.0–28.58)	12.3 (7.9–23.01)

Data presented as mean ± SD; parameters marked # indicate median (range); * denotes significance compared to normal weight group; ¶denotes significance compared to normal and over weight groups. ¶*P < 0.05

**Table 2:** Spearman's correlation analyses using BMI as dependent variable

	Normal		Overweight		Obese	
	R	P-Value	R	P-Value	R	P-Value
Waist circumference	0.04	0.90	0.25	0.17	0.39	0.01
Hip circumference	0.22	0.45	0.15	0.41	0.50	0.001
CRP	0.21	0.35	0.15	0.40	0.43	0.003

Parameters that were not included were insignificant

In a stepwise multiple linear regression analysis, using IL-6 as the dependent variable and all other parameters as independent variables, leptin was found to be a significant predictor among the overweight group (R = 0.75; p < 0.0001) and TGF-α, CRP and BMI for the normal weight group (R = 0.64; p < 0.001) (Table 3). IL-6 was the sole significant predictor for the levels of TGF-α among the normal weight group (R = 0.36; p = 0.003) (Table 3). Adiponectin levels, hip and waist circumferences were significant predictor of BMI in normal weight group (Table 3). In addition to these parameters, CRP was found to be an additional predictor of BMI in obese group (Table 3). Levels of insulin, glucose and diastolic pressure were significant predictors of HOMA-IR in normal and obese weight groups (Table 3), while insulin, glucose, LDL-cholesterol and waist circumference were significant predictors in over weight group (Table 3).

**Table 3:** Significant predictors of breast cancer risk using dependent variables of interest

	**Dependent Variables**			
**Group**	**IL-6**	**TGF-α**	**BMI**	**HOMA-IR**

**Normal weight**	TGF-α, CRP, BMI R = 0.64; p < 0.001	IL-6 R = 0.35 p < 0.009	Adiponectin, Hip, waist R = 0.88; p < 0.001	Insulin, glucose, diastolic, R = 0.97; p < 0.001

**Overweight**	Leptin, R = 0.75; p < 0.0001	Triglycerides R = 0.44 P < 0.046	------	Insulin, glucose, LDL, waist R = 0.98; p < 0.001

**Obese**	------	------	Hip, waist, CRP, adiponectin R = 0.84; p = < 0.001	Insulin, glucose, diastolic, R = 0.97; p < 0.001

Independent variables entered: BMI, waist circumference, hip circumference, WHR,

leptin, adiponectin, CRP, TNF-α, insulin, HOMA-IR, TGF-α, and IL-6

Obesity is increasingly associated with postmenopausal breast cancer risk, whereas, in premenopausal women there is an inverse relationship between BMI and the risk [31,32]. However, both pre-menopausal and post menopausal breast cancer patients who are obese are more likely to have a poor final out come than those who are non-obese [32]. It is widely accepted that the influence of obesity on risk of breast cancer specifically applies to upper body obesity. Consistently, increased waist to hip ratio and waist circumference has shown to be associated with the breast cancer risk which, importantly was slightly diminished by controlling for the BMI [33]. This suggests that increased waist to hip ratio and waist circumference play critical role in the breast cancer risk than increased BMI alone. In the present study, the significant increase in waist and hip circumference in obese group in comparison to normal weight group and linear correlation between BMI and the waist and hip circumference in the obese group suggest that the women with increased upper body obesity are at an increased risk for breast cancer. This was further strengthened by the lack of significant correlations between normal body weight group and overweight group, despite a significant increase in BMI in the later.

Diabetic condition induces changes in several hormonal systems, including insulin, insulin like growth factors, estrogens and other cytokines that may affect the breast cancer risk. Characteristics of T2DM including insulin resistance and the resultant hyperinsulinemia are strongly correlated with postmenopausal as well as pre-menopausal breast cancer risk [6,7]. Hyperglycemia, which develops as a result of resistance to the action of insulin, is positively associated with the elevated levels of glucose. High fasting glucose levels were directly correlated with breast cancer risk both in pre-menopausal and postmenopausal women [7,34]. Studies also indicate that fasting glucose levels ≥ 126 mg/dl, which is cutoff for defining the T2DM were related to an increased risk for the carcinogenesis of the breast [34,35]. In addition, reduced HDL-cholesterol and increased blood pressure have contributed to increased risk for breast cancer [26,36]. Thus, type 2 diabetic status, with its multiple risk factors, appears to be an important contributor of breast cancer risk. In our study, though there is a lack of correlation between the breast cancer risk factors, including cholesterol, insulin, glucose and insulin resistance, with an increasing BMI, the presence of diabetic status in the studied subjects itself may contribute to increased breast cancer risk. This can be attributed to above the normal cutoff values of these parameters in all the subjects irrespective of their BMIs; for example, increased glucose levels in type 2 diabetics may still pose a significant breast cancer risk in women with the normal body weight. This might also explain a causal relationship for the presence of breast cancer risk in the pre-menopausal women in the present study.

Obesity and T2DM also have in common an increased production of leptin and a decreased production of adiponectin [9,10]. Such an association is consistent with a positive impact of both obesity and T2DM on breast cancer risk [11,12]. In addition, a linear correlation between the plasma CRP and leptin concentrations contributes to an enhanced breast cancer risk [18,37]. Accordingly, decreased adiponectin levels in obese as well as overweight subjects and increased leptin levels in obese women in this study are consistent with the association of these adipocytokines with obesity and T2DM and thus suggest increased breast cancer risk. Presence of diabetic status, irrespective of BMI itself indicates breast cancer risk in the studied subjects owing to above or below the normal cutoff values of leptin and adiponectin levels respectively. This is in agreement with the finding that leptin levels in T2DM are positively correlated with the degree of insulin resistance independent of BMI and body fat content [38].

Inflammation is associated with poor prognosis and decreased survival in many cancers. Obesity per se is considered a subclinical inflammation and thus, as markers of inflammation and their positive role in breast carcinogenesis, the increased levels of IL-6 and CRP in obese women in the present study are consistent with the breast cancer risk. Importantly, in obese women, a positive correlation between IL-6 and waist circumference, which itself is an independent risk factor for breast cancer, suggests obesity induced inflammatory response and enhanced risk for carcinogenesis. In addition, CRP is positively correlated with leptin and inversely with the adiponectin levels [18,19]. Thus, in obesity, the adipocytokines and the inflammatory mediators might exert an additive effect to positively impact the breast cancer etiology. Unaltered TNF-α in the studied subjects may relate its dependency on the insulin as the TNF-α secretion was positively correlated with the plasma insulin concentrations [39], which is consistent with unaltered serum insulin levels in the subjects independent of BMI. Lack of change in TGF-α in the studied subjects may possibly indicate its association at a later stage in the disease progression or exclusively associated with postmenopausal breast cancer risk. Though, T2DM and obesity induced breast cancer risk found in the premenopausal women in this study may contradicts with a number of studies, while considering that the etiological factors for breast cancer act over a long time, menopausal transition may unlikely have a significant impact in altering the cancer risk. Alternatively, factors responsible for the disease risk may differ between pre and post-menopausal states.

We acknowledge several limitations in this study. Due to small sample size especially in normal and overweight groups, some of the associations might be altered if analyzed on a larger sample size. Though, the presence of diabetic status in the studied subjects itself was found to be a risk factor for breast cancer in our study, we did not verify this finding by comparing with non diabetic normal control subjects. Additionally, conclusions made in this study were based on the prevalence of breast cancer risk factors in obese and T2DM subjects, while not directly evaluating these risk factors in the breast cancer patients. Due to cross sectional nature of this study and small sample size the observations need to be followed up with much larger sample size and in a prospective setup to make a causal inference.

## Conclusion

In summary, we found the presence of increased breast cancer risk in obese type 2 diabetic premenopausal women. Furthermore, altered expression of inflammatory mediators and the adipocytokines in obese diabetic women may collectively promote the breast cancer etiology as compared to women with normal body weight. Since the association of adipocytokines and inflammatory markers with breast cancer has been firmly established, our objective to measure these parameters in obese T2DM subjects while not in breast cancer cases was to assess whether the existence of these metabolic abnormalities confers an additional risk to develop the breast cancer. In this context, findings of this study attain significance in predicting the breast cancer risk and avoiding its carcinogenesis. Additionally, this study recommend a healthy life style, regular physical activities and a healthy diet to counter the growing epidemics of obesity, T2DM and associated disorders such as breast cancer and cardiovascular diseases.

## Competing interests

The authors declare that they have no competing interests.

## Authors' contributions

NMA; designed and conceived the study, participated in preparing the draft. MSA; carried out the biochemical measurements and obtained the data, participated in statistical analysis. OSA; Helped design and conceive the study, critically evaluated the draft. TH; Analyzed and interpreted the data, drafted the manuscript, participated in biochemical measurements. All authors read and approved the final manuscript.
